# Temperature-Dependent Structural Transition in Cu-Intercalated Trigonal CuYbSe_2_

**DOI:** 10.1021/acs.chemmater.5c02470

**Published:** 2025-12-02

**Authors:** Matt Boswell, Mingyu Xu, Saban M. Hus, Antonio M. dos Santos, Weiwei Xie

**Affiliations:** † Department of Chemistry, 3078 Michigan State University, East Lansing, Michigan 48824, USA; ‡ Neutron Scattering Division, 6146 Oak Ridge National Laboratory, Oak Ridge, Tennessee 37831, USA; § Center for Nanophase Materials Sciences, Oak Ridge National Laboratory, Oak Ridge, Tennessee 37831, USA

## Abstract

Rare-earth delafossites, ARCh_2_; A = alkali metal, R = rare-earth, Ch = chalcogen which consist of intercalated rare-earth metal dichalcogenides, host frustrated triangular lattices that are fertile ground for exotic phenomena. In most cases, the triangular rare-earth sublattice arises from *R*-3*m* (No. 166) structures with three layers of rare-earth metal dichalcogenide octahedra or *P*6_3_/*mmc* (No. 194) structures with two such layers, analogous to those found in transition metal dichalcogenides. Substituting the alkali metal with Cu^+^ yields a distinct trigonal crystal symmetry—*P*-3*m*1 (No. 164)—in these structures. This symmetry change alters the coordination environment from ASe_6_ octahedra in *R*-3*m* AYbSe_2_ to CuSe_4_ tetrahedra in CuYbSe_2_, resulting in shortened rare-earth to rare-earth separations and significantly reduced interlayer distances. Using X-ray single-crystal diffraction, powder neutron diffraction, resistance, and specific heat measurements, a structural transition slightly below room temperature (258 K) is observed. The low-temperature structure is a lower-symmetry *I*2/*m* structure, accompanied by partial Cu-site vacancy ordering. The combination of Cu disorder and the triangular lattice geometry in CuYbSe_2_ provides a promising platform for investigating frustrated magnetism and unconventional transport phenomena.

## Graphical abstract

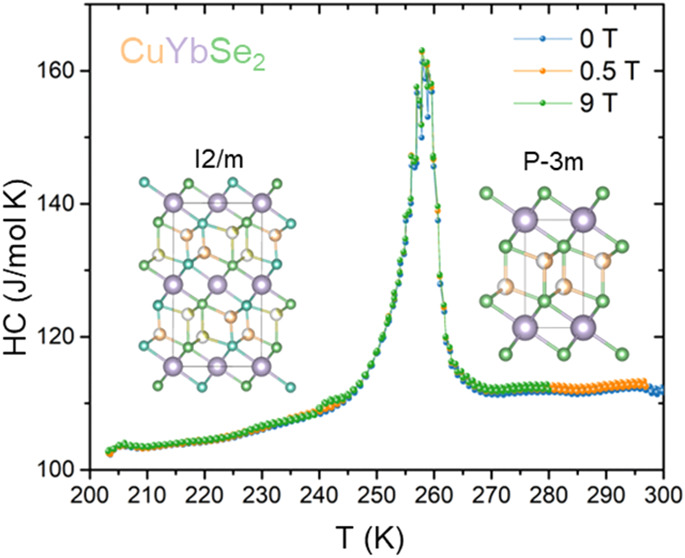

## Introduction

Chalcogenides, comprising chalcogen elements (S, Se, or Te) in combination with one or more electropositive elements such as transition metals, rare-earth elements, or main group elements, display a broad spectrum of functional properties that underpin applications in thermoelectrics, superconductivity, optoelectronics, and catalysis. Exemplary systems include bulk thermoelectric Cu_2–*x*_Se and SnSe, and the superconducting Fe_1+*x*_Se.^1–3^ Rational synthetic control over chalcogenides - particularly for thermoelectric applications—has been advanced substantially by the work of Kanatzidis and co-workers, inspiring numerous studies, including the present investigation on the thermal evolution upon cooling of the structure of 1T-CuYbSe_2_.^4^

Rare-earth delafossites (ARCh_2_; A = alkali metal, R = rare-earth element, Ch = chalcogen) have garnered significant attention owing to their geometric—and the consequent—magnetic frustration, layered architectures, and structural parallels to transition metal dichalcogenides (TMDs). In both families, two-dimensional (2D) layers of metal-chalcogen polyhedra are the key structural motif. In TMDs, these layers are held together by van der Waals forces, whereas in ARCh_2_ compounds they are bridged by intercalated alkali cations.^5^ TMDs exhibit multiple polytypes, including 1T (*P*-3*m*1), 2H (*P*6_3_/*mmc*), and 3R (*R*-3*m*), with the structure dictated by the specific metal-chalcogen chemistry, yielding diverse physical properties.^6^ In contrast, ARCh_2_ compounds predominantly adopt the 3R or 2H structures shown in Figure 1, with Yb^3+^-based members largely restricted to the 3R type.^7^ In these compounds, the Yb^3+^ adopts a *S*_eff_ = 1/2, *J*_eff_ = 1/2, supporting strong quantum fluctuations. Yb-based ARCh_2_ materials are therefore appealing candidates for quantum spin liquids (QSL), demonstrated by NaYbSe_2_.^8,9^ Chemical substitution of the alkali metal tunes structural parameters and can modulate proximity to quantum criticality: for example, CsYbSe_2_ lies further from the quantum critical regime than NaYbSe_2_.^10,11^ LiYbSe_2_, because of the small ionic radius of Li^+^, adopts a three-dimensional pyrochlore (*Fd*-3*m*) structure, leaving its position in the QSL phase diagram unclear.^12^

**Figure 1:**
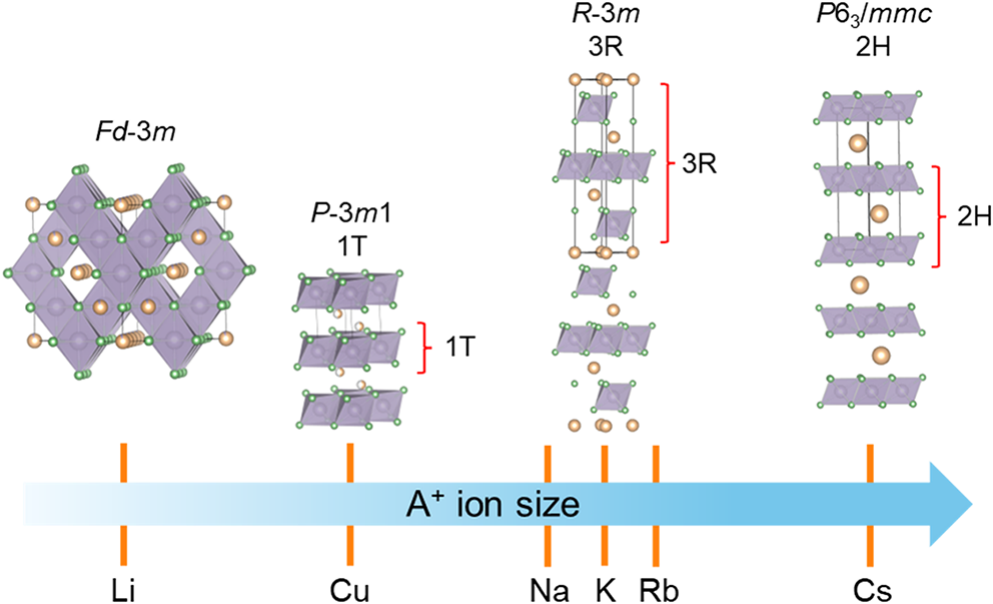
Crystal structures of AYbSe_2_ with progressively increasing A^+^ ionic radius, showing the unit cells of the 1T, 3R, and 2H polytypes. The diagrams highlight the distinct stacking sequences and coordination environments of the YbSe_6_ octahedra in each structure.

Replacement of the alkali metal with monovalent Cu^+^, isoelectronic and similar in ionic radius to Li^+^, stabilizes the 1T polytype (*P*-3*m*1) in Yb-based delafossites.^13^ This access to all the structures observed layered TMD-like polytypes offers a unique avenue for tuning structural dimensionality and emergent quantum states in rare-earth chalcogenides. Here, we report the temperature-dependent structural phase transition of 1T-CuYbSe_2_, illustrating the structural diversity and functional potential of rare-earth delafossites.

## Experimental Section

### Chemical Synthesis of CuYbSe2

Large (several mm^3^) crystal of CuYbSe_2_ with a black metallic shine appearance were obtained by a self-flux reaction of the pure elements. Stoichiometric ratios of Cu (Alfa Aesar 99.9%) and Yb (Alfa Aesar 99.9%) were mixed under a 4-fold excess of Se (ThermoScientific 99+%) (CuYbSe_2_; 1:1:4). The resultant mixture of elements was placed in an alumina crucible, which was then sealed inside a quartz tube flushed with argon to ensure no oxygen contamination. The ampule was sealed under vacuum and flushed with argon. The sample was heated to 1150 °C at a rate of 30 °C/h where it remained for 4 h. The sample was then cooled to 600 °C at a rate of 1 °C/h. Once reaching 600 °C the sample was removed and quenched with water. To ensure no Se vacancies were present, because of its high volatility, excess Se was used and acted as the flux. Sample synthesis was slightly modified from Daszkiewicz.^13^

### Single Crystal X-ray Diffraction

The crystal structures and chemical compositions of all synthesized crystals were confirmed through single crystal X-ray diffraction (SC-XRD) analysis. The crystals were meticulously mounted on a Kapton loop and examined using a Rigaku Synergy-S diffractometer, operating with Mo Kα radiation (λ = 0.7107 Å). The resulting diffraction data were processed using Olex2 software for precise structural determination and refinement.^14^ Temperature dependent single crystal measurements were measured in 20 K increments down to 100 K with liquid nitrogen.

### Powder X-ray Diffraction (PXRD)

The phase purity of the samples was evaluated using powder X-ray diffraction. PXRD measurements were performed on a benchtop Rigaku MiniFlex 600 diffractometer, utilizing Cu Kα radiation (λ = 1.5406 Å). Data were collected over a Bragg angle (2θ) range from 10° to 90° with a step size of 0.005° and a scan speed of 1°/s. Rietveld refinement of PXRD patterns was conducted using GSAS-II software to accurately assess the phase purity.^15^

### Neutron Powder Diffraction

Neutron diffraction measurements were taken at SNAP (beamline 3) of the Spallation Neutron Source (SNS) at the Oak Ridge National Laboratory (ORNL). CuYbSe_2_ crystals were finely ground to a powder and approximately 550 mg was loaded into a single toroidal Paris-Edinburgh press without pressure medium. Approximately 5 mg of lead foil was used to act as a pressure calibrant. Detectors were set at 90° and 50° to capture high resolution and high *d*-spacing or CuYbSe_2_. Rietveld refinement of the powder pattern was carried out with the GSAS-II software.

### X-ray Photoelectron Spectroscopy (XPS)

X-ray photoelectron spectroscopy was performed at room temperature using a SPECS Focus 500 monochromated Al Kα X-ray source and PHOIBOS-150 hemispherical analyzer operated in the medium area mode and 40 eV pass energy. Energies were calibrated using the Ag Fermi edge core levels. Single crystals of CuYbSe2 were mounted on a Mo sample holder with silver-based epoxy. XPS peak positions were also checked by using C 1s line from adventitious carbon as a reference which did not show any significant shift with and without charge neutralization.

### Heat Capacity and Resistance Measurements

Temperature-dependent resistance measurements, as well as temperature-dependent specific heat measurements, were carried out using Quantum Design (QD), Physical Property Measurement System (DynaCool). The specific heat measurements were carried out using the relaxation technique as implemented in the Heat Capacity option of the PPMS. The DC electrical resistance was measured in a standard four-contact geometry with a constant current of 0.1 mA. Platinum wires (50 μm diameter) were bonded to the sample using silver paint (DuPont 4929N). The magnetic field, up to 90 kOe, was applied along the *c* axis, perpendicular to the current in the *ab* plane.

## Results and Discussion

CuYbSe_2_ crystallizes in the 1T-type structure with space group *P*-3*m*1. Similar to other ARCh_2_ compounds, CuYbSe_2_ features octahedrally coordinated Yb^3+^ ions bonded to Se^2–^ anions, separated by half-occupied layers of Cu^+^ ions, as illustrated in Figure 2a. Free refinement of elemental occupancies yields a composition of Cu_1.08_Yb_0.98_Se_2_, consistent with the compositional variability reported for this system. Indeed, different synthesis routes have produced a range of stoichiometries from the charge-balanced CuYbSe_2_ to Cu_1.14_YbSe_2.16_.^16,17^ Such variations in Cu content may strongly influence the low-temperature magnetic dynamics of this disordered and frustrated lattice. The cation disorder in CuYbSe_2_ resembles that in YbMgGaO_4_, a compound initially proposed to host a quantum spin liquid (QSL) ground state. Subsequent studies, however, suggested that random site disorder between Yb^3+^ layers could drive random-singlet formation, mimicking QSL-like behavior.^18,19^ Despite these findings, several reports still argue for a robust QSL state persisting in the presence of disorder.^20^ Whether the ground state of YbMgGaO_4_ is a true QSL or a random-singlet system, it is evident that structural disorder critically impacts the resulting magnetic ground state and thus demands careful examination. By analogy, CuYbSe_2_ likely exhibits a random-singlet or possibly a spin-glass ground state, depending on the extent of Cu/Yb site disorder.^16,21^ For consistency, the composition Cu_1.08_Yb_0.98_Se_2_ will hereafter be referred to simply as CuYbSe_2_. To disentangle the disorder-driven effects from intrinsic quantum phenomena, systematic measurements are required.^18,19^ Structurally, the interlayer Yb–Yb distance (*J_c_*) in CuYbSe_2_ is shorter than that in NaYbSe_2_, where Yb^3+^ ions are staggered between triangular layers (Table 1).^8,22^ This reduced interlayer spacing, together with comparable in-plane next-nearest-neighbor exchange (*J*_2_), renders CuYbSe_2_ distinct among ARCh_2_ compounds. Such connectivity may promote three-dimensional magnetic interactions, suggesting that CuYbSe_2_ is better described by an anisotropic Heisenberg model rather than the conventional *J*_1_–*J*_2_ triangular lattice model typically used for AYbCh_2_ systems in QSL analyses.^9,23^

**Figure 2:**
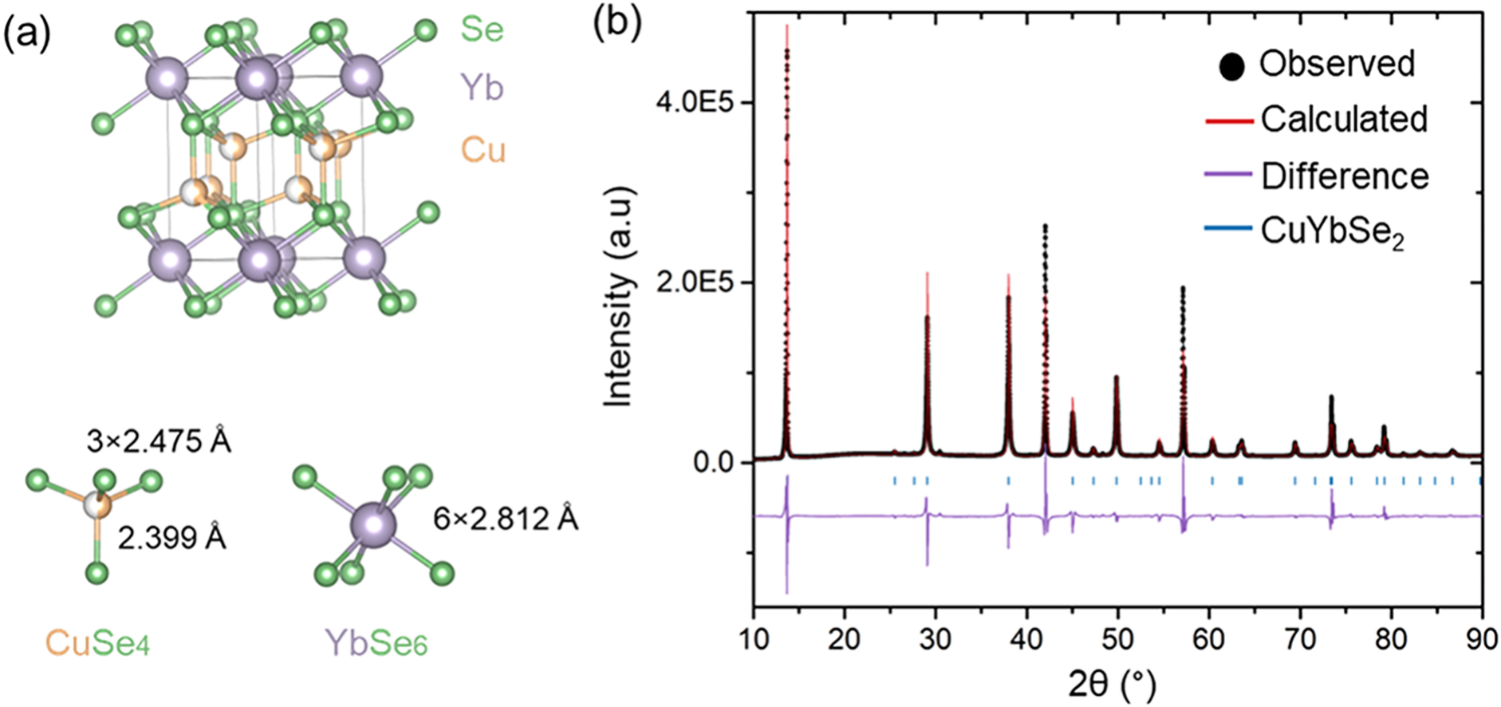
(a) Crystal structure of CuYbSe_2_ in the *P*-3*m*1 space group, showing YbSe_6_ octahedra and CuSe_4_ tetrahedra within the layered framework. (b) Rietveld refinement of the powder X-ray diffraction data for CuYbSe_2_, confirming phase purity and structural consistency with the *P*-3*m*1 model.

**Table 1:** Comparative Yb^3+^-Yb^3+^ Distances in CuYbSe_2_ (*P*-3*m*1) and NaYbSe_2_ (*R*-3*m*), Highlighting Differences in Interlayer (*J_c_*) and In-Plane Next-Nearest-Neighbor (*J*_2_) Separations

	CuYbSe_2_	NaYbSe_2_
***J***_**1**_	4.0167(15) Å	4.0568(9) Å
***J***_**2**_	6.9571(6) Å	7.0266(10) Å
***J**_**c**_*	6.429(1) Å	7.309(2) Å

Another notable distinction of CuYbSe_2_ lies in its crystal appearance. While most ARCh_2_ materials are light-colored and transparent, consistent with their Mott-insulating or charge-transfer insulating nature, CuYbSe_2_ forms black crystals with a metallic luster. Despite this appearance, resistance measurements reveal a high resistance exceeding 5000 Ω, characteristic of an insulating or semiconducting material. The low electrical conductivity coupled with a dark metallic color suggests the presence of strong optical absorption, likely associated with structural defects or localized electronic states within the band gap. A relevant comparison can be drawn with oxygen-deficient TiO_2_, which also exhibits a black, yet insulating appearance. In TiO_2_, removal of oxygen reduces Ti^4+^ to Ti^3+^, introducing localized defect states that absorb visible light while maintaining low electrical conductivity.^24^ Although CuYbSe_2_ may exhibit analogous defect-induced absorption, the underlying mechanism is likely distinct, potentially linked to Cu-site vacancies or mixed valence states that perturb the local electronic environment. SEM-EDS analysis (Figure S1) confirms that the elemental ratios of Cu, Yb, and Se are close to the ideal stoichiometric composition of CuYbSe_2_, indicating that the observed optical behavior is intrinsic to the crystal structure rather than due to significant compositional deviation.

To determine the crystal structure of CuYbSe_2_ across a broad temperature range and to identify potential structural phase transitions, temperature-dependent single-crystal X-ray diffraction (SCXRD) measurements were performed from 300 to 100 K at nine discrete temperature points using the same crystal. The diffraction data were refined using both the trigonal *P*-3*m*1 and monoclinic *I*2/*m* structural models, with the refinement results summarized in Tables S1 and S2. Throughout the entire temperature range, the refined *R*-values were consistently lower for the *P*-3*m*1 model than for *I*2/*m*, suggesting that the trigonal structure provides a better overall fit. However, the residual electron density peaks and holes were notably larger for the *P*-3*m*1 refinements, indicating potential local structural distortions or disorder not fully captured by this symmetry. Consequently, the space-group assignment could not be unambiguously resolved based on X-ray diffraction alone, likely due to the presence of site disorder or subtle symmetry breaking within the Cu layers.

To further elucidate the structural evolution, temperature-dependent neutron diffraction was employed. While the room-temperature structure agrees well with previously reported *P*-3*m*1 models, the neutron diffraction data reveal the onset of additional Bragg reflections below ∼250 K, inconsistent with the trigonal symmetry (Figure 3). These five extra peaks can be indexed by a monoclinic *I*2/*m* unit cell with lattice parameters *a* = 6.940(5) Å, *b* = 4.030(3) Å, *c* = 12.900(5) Å, and β = 90.14(8)°, which remains stable down to 80 K, the lowest measured temperature. The monoclinic phase can be described as a subtle distortion from the parent trigonal framework, with no significant unit-cell volume change beyond the expected thermal contraction. The refined neutron diffraction pattern of the *I*2/*m* phase at 80 K is shown in Figure 4b, and the corresponding crystallographic parameters for both the standard setting (*C*2/*m*) and monoclinic *I*2/*m* structures are listed in Tables S5 and S6.

**Figure 3:**
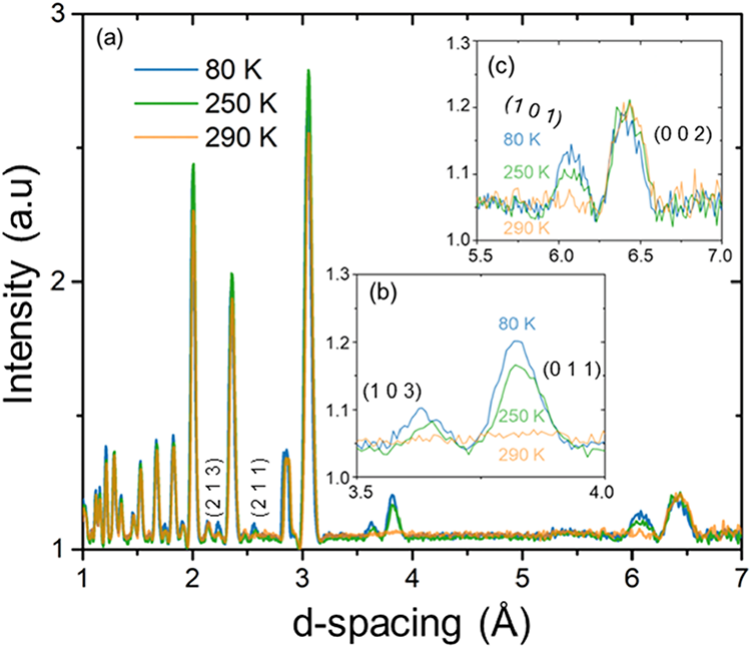
(a) Temperature dependence of the neutron powder diffraction of CuYbSe_2_ at ambient conditions. (b) Additional peaks caused by the structural distortion. Two peaks emerge between 3.5 and 4.0 Å with the 250 K peaks occurring with lower intensity. (c) Additional peak from the monoclinic structure around 6.0 Å. Note that the 250 K, which is right at the transition, is less intense.

**Figure 4:**
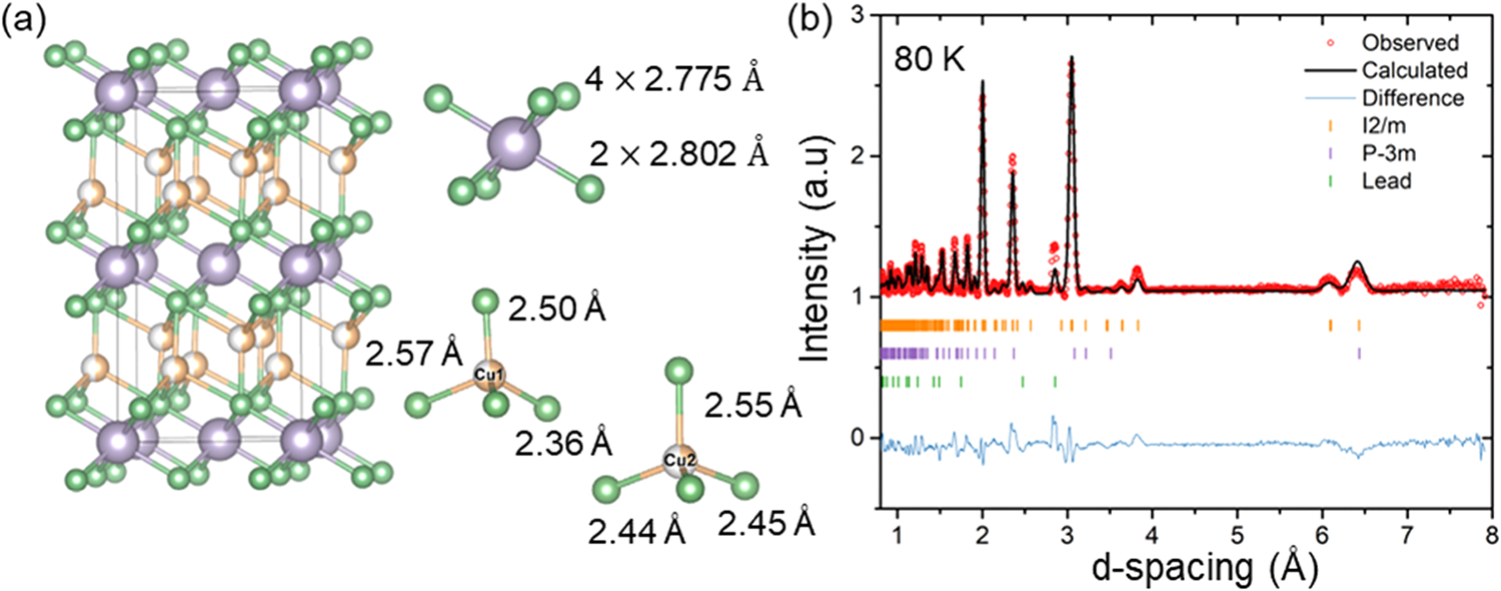
(a) Comparison between the low-temperature monoclinic *I*2/*m* structure and the high-temperature *P*-3*m*1 parent structure of CuYbSe_2_, highlighting the distinct CuSe_4_ tetrahedra and YbSe_6_ octahedra. (b) Rietveld refinement of the neutron powder diffraction data collected for the monoclinic *I*2/*m* phase of CuYbSe_2_.

The Yb^3+^ triangular lattice is retained in the monoclinic structure, although the local ligand environment undergoes significant modification. The loss of trigonal symmetry causes the selenium atoms to split into two distinct crystallographic sites, Se3 and Se4, resulting in inequivalent Yb–Se bond lengths. In the parent *P*-3*m*1 structure, the Yb–Se distance is 2.8159(7) Å. In contrast, in the monoclinic phase, four Yb–Se4 bonds shorten to 2.775(4) Å, while the two remaining Yb–Se3 bonds are slightly longer at 2.802(6) Å. Inspection of the distorted CuSe_4_ tetrahedra in the parent structure can be qualitatively described using an “umbrella” analogy, in which the Cu–Se “rib” bonds measure 2.475(1) Å, and the shorter “handle” bond measures 2.409(5) Å. Upon transition to the monoclinic phase, Cu atoms shift from ideal tetrahedral coordination to distorted tetrahedral environments, now occupying two independent sites (Cu1 and Cu2), as shown in Figure 4a. These copper sites differ in their Cu–Se and Cu–Yb interatomic distances (summarized in Table 2). Notably, Cu1 exhibits shorter Cu–Se bonds and a larger Cu–Yb separation compared to Cu2. In the parent *P*-3*m*1 structure, the Cu–Cu distance is 2.794(6) Å. In the monoclinic phase, the Cu1–Cu1 and Cu2–Cu2 separations increase to 3.0013(13) Å and 3.0348(13) Å, respectively, while the Cu1–Cu2 distance decreases to 2.5299(16) Å. The *I*2/*m* space group was selected to preserve the same *c*-axis as the trigonal parent; however, refinements in the standard *C*2/*m* setting yield comparably good agreement. In the monoclinic phase, the honeycomb symmetry formed by Cu and Se atoms is disrupted, leading to the loss of 3-fold rotational symmetry (Figure S3). A symmetry-mode analysis using ISODISTORT^25,26^ reveals that the structural transition from the parent to the child phase corresponds to a Γ-point distortion, involving both lattice strain (Γ_1_^+^) and in-plane symmetry breaking (Γ_3_^+^). The child structure retains the same translational periodicity as the parent, with the symmetry reduction driven primarily by internal atomic displacements.

**Table 2:** Interatomic Distances within the CuSe_4_ Tetrahedra and Their Nearest-Neighbor Yb^3+^ Ions in the Monoclinic *I*2/*m* Phase of CuYbSe_2_

Cu1-Handle	Cu1–Se3	Cu1–Se4	Cu2-Handle	Cu2–Se3	Cu2–Se4	Cu1–Yb	Cu2–Yb
2.502(1)Å	2.569(1) Å	2.355(2) Å	2.546(1) Å	2.443(2) Å	2.449(1) Å	3.306(1) Å	3.241(1) Å

An assignment of the preferred Cu site was attempted based on powder neutron diffraction refinements; however, the results remained inconclusive. Starting from a 50:50 occupancy model, the Cu2 site appeared slightly favored over Cu1. When the initial ratios were adjusted to favor Cu1 (70:30), the refinements converged to an equally satisfactory fit, suggesting that either site can be modeled as the preferred site. This indicates that Cu and Se atoms shift cooperatively to accommodate variations in site occupancy. Initial refinements assuming full Cu occupancy (1.0 stoichiometric ratio) yielded an approximate 70:30 distribution between the two sites. Increasing the total Cu occupancy to 1.2, which is consistent with single-crystal X-ray diffraction results, led to a broader disparity, favoring Cu2 with a 90:30 ratio. When occupancy constraints were completely removed, the refinements converged to similar values, further supporting the dominance of the Cu2 site. Notably, the preferred Cu2 position lies closer to the Yb^3+^ ions and exhibits slightly longer Cu–Se bond distances. The fact that the powder pattern can be refined using two closely related structural models suggests that the distortion is not fully coherent and may vary between layers. This behavior resembles a Peierls-type distortion involving a doubling along the *c*-axis, consistent with the hysteresis observed in resistance measurements.^27,28^ Additionally, as observed in other Yb^3+^-transition-metal systems, the valence of Yb^3+^ may exhibit temperature-dependent reduction toward Yb^2+^, promoting magnetic inhomogeneity and potentially driving spin-glass behavior. As illustrated in Figure S3, the view along the *c*-axis highlights both the similarities and subtle differences between the two structures, where the shifted Cu ions disrupt the ideal puckered honeycomb lattice of the parent *P*-3*m*1 phase.

Evidence for the structural phase transition is further supported by temperature-dependent heat capacity and electrical resistance measurements. In the heat capacity data, a pronounced anomaly centered near 255 K exhibits characteristics consistent with a second-order transition (Figure 5). Although relatively sharp, the anomaly extends over a broad temperature range (∼245–264 K) and displays a monotonic evolution in total entropy. After subtracting the lattice contribution and integrating the excess heat capacity, the associated entropy change is estimated to be ∼1.2 J/mol-K. The transition is nonmagnetic in origin, as the application of a 9 T magnetic field does not significantly alter the magnitude or position of the anomaly. The heat capacity behavior of CuYbSe_2_ closely resembles that of Cu_2_Se, which exhibits a similar second-order-like transition, in contrast to Cu_2_S, whose much sharper feature is characteristic of a first-order transition.^1^ However, the exact nature of the transition in Cu_2_Se remains controversial; some studies have suggested first-order character due to coexistence of multiple phases within nanoscale grain boundaries.^29^ A comparable ambiguity is observed in CuYbSe_2_, as evidenced by a hysteresis in the resistance data (Figure 6), a hallmark of first-order behavior. These observations suggest that the transition in CuYbSe_2_ may possess hybrid character, incorporating both first- and second-order features. Structurally, the monoclinic and hexagonal phases of CuYbSe_2_ are closely related, differing primarily by subtle lattice distortions. Nevertheless, this minor structural perturbation produces a substantial heat capacity change, implying additional underlying effects such as modifications in the electronic band structure, enhanced electron–phonon coupling, or orbital ordering. The large anomaly is reminiscent of the ionic order–disorder transition reported in Cu_2_Se.^1,30^ The low-temperature phase of Cu_2_Se has been proposed to consist of several energetically comparable structural configurations, as suggested by Qiu et al.^29^ The structural assignment of the low-temperature Cu_2_Se phase remains highly debated, with monoclinic, orthorhombic, and triclinic models all proposed.^31–33^ Determining the precise structure has been challenging for several reasons. First, obtaining high-quality single crystals that avoid severe twinning upon cooling from the high-temperature cubic phase has proven difficult.^34^ Additionally, diffuse scattering from disordered Cu ions complicates refinement, as analyses based solely on intense Bragg reflections yield an average *R*-3*m* symmetry accompanied by several proposed superstructures.^35^ Roth and Iversen later argued that these apparent superstructures arise from two-dimensional ordering that becomes disordered along the third dimension.^34^ This interpretation was supported by three-dimensional pair distribution function analysis, which revealed a periodic local ordering combined with stacking disorder. Overall, while the average structure at low temperature retains nominal *R*-3*m* symmetry, the diffuse scattering features clearly indicate local deviations from this high-symmetry model.^34^

**Figure 5:**
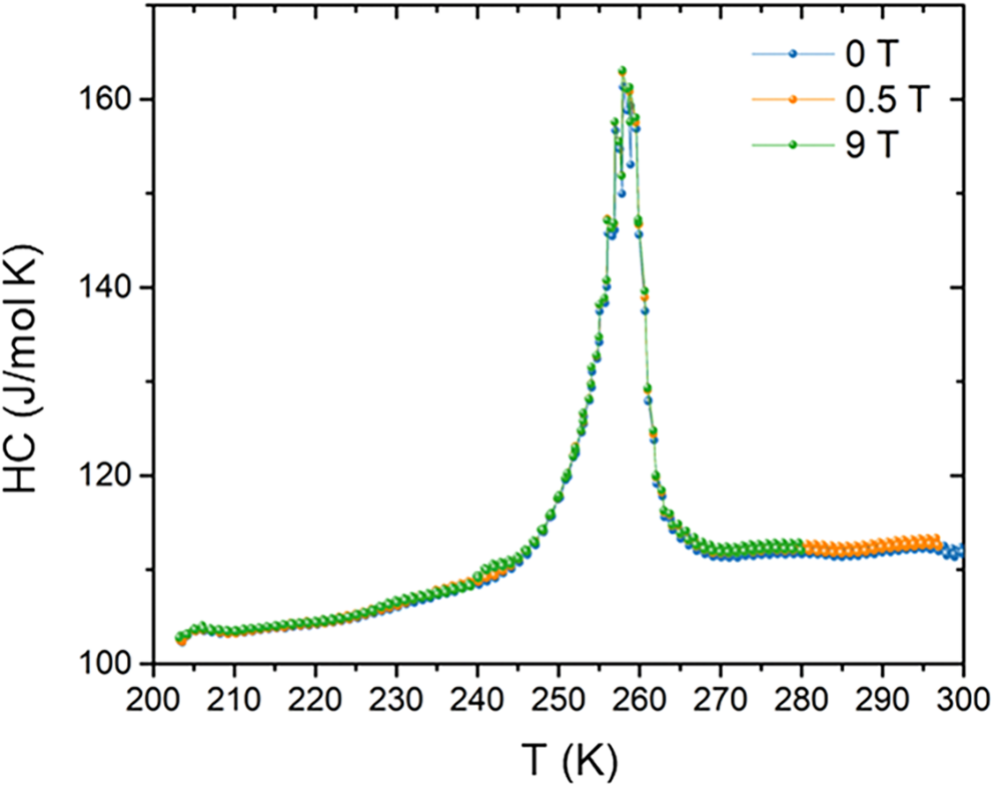
Temperature-dependent heat capacity of CuYbSe_2_ measured from 203 to 300 K under applied magnetic fields of 0, 0.5, and 9 T.

**Figure 6:**
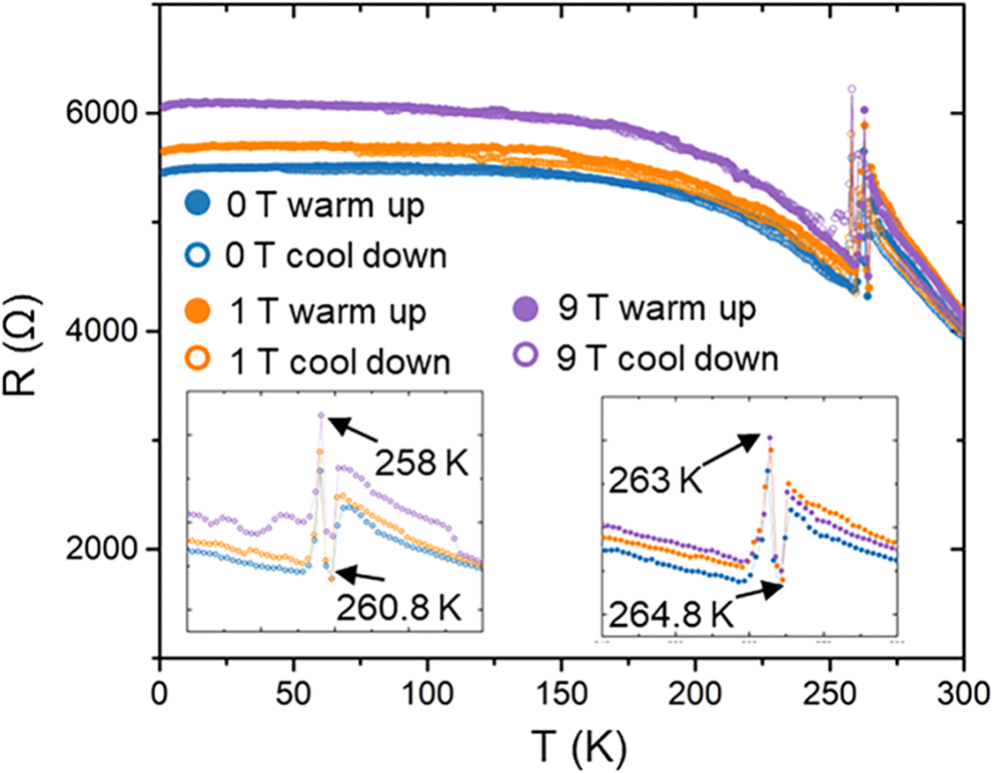
Temperature-dependent electrical resistance (ρ) of CuYbSe_2_ measured from 2 to 300 K under applied magnetic fields of 0, 1, and 9 T.

The observed structural disorder provides insight into why CuYbSe_2_ exhibits both optical absorption and electrical insulation. The additional degrees of freedom associated with the Cu and Se sublattices introduce local distortions and defect states that can generate electronic levels accessible for photoexcitation. Similar behavior has been reported in Cu_2_S, where the closed-shell configuration of Cu^+^ and its relatively soft bonding with chalcogen atoms permit enhanced cation mobility within the lattice.^36^ A more direct comparison can be made with Cu_2_Se, which undergoes a monoclinic-to-antifluorite phase transition above 400 K. In the high-temperature antifluorite phase, Cu Se becomes superionic, exhibiting substantial Cu-ion mobility persisting down to ∼100 K.^1^ Although Cu_2_Se and CuYbSe_2_ differ crystallographically, the analogy suggests a related mechanism of Cu-ion dynamics in CuYbSe_2_. In Cu_2_Se, Cu^+^ mobility arises primarily from hopping between octahedral and tetrahedral sites.^31^ In contrast, CuYbSe_2_ contains partially vacant Cu tetrahedral sites, providing potential pathways for hopping between occupied and vacant sites. Notably, in Cu_2_Se, introducing Cu vacancies enhances superionicity, Cu_1.98_Se displays greater ionic mobility than stoichiometric Cu_2_Se, and this trend continues in Cu_1.75_Se, which retains superionic behavior to even lower temperatures.^37^ At ambient temperature, the Cu ions in CuYbSe_2_ appear distributed approximately equally among their crystallographic sites; however, upon cooling, one site becomes energetically favored, resulting in local distortion and symmetry lowering. Indirect evidence for Cu mobility in CuYbSe_2_ is provided by X-ray photoelectron spectroscopy (XPS) measured at room temperature. The characteristic Cu^+^ 2p_3/2_ peak typically appears near 933 eV, yet in CuYbSe_2_ it shifts to a lower binding energy of 931.8 eV (Figure 7). Such reductions of 1–2 eV are commonly observed in superionic conductors, reflecting the loosely bound nature of mobile ions and their enhanced ability to polarize the photoemission hole.^38^ The extent and mechanism of Cu-ion hopping and long-range propagation within the CuYbSe_2_ lattice, however, remain open questions warranting further investigation.

**Figure 7:**
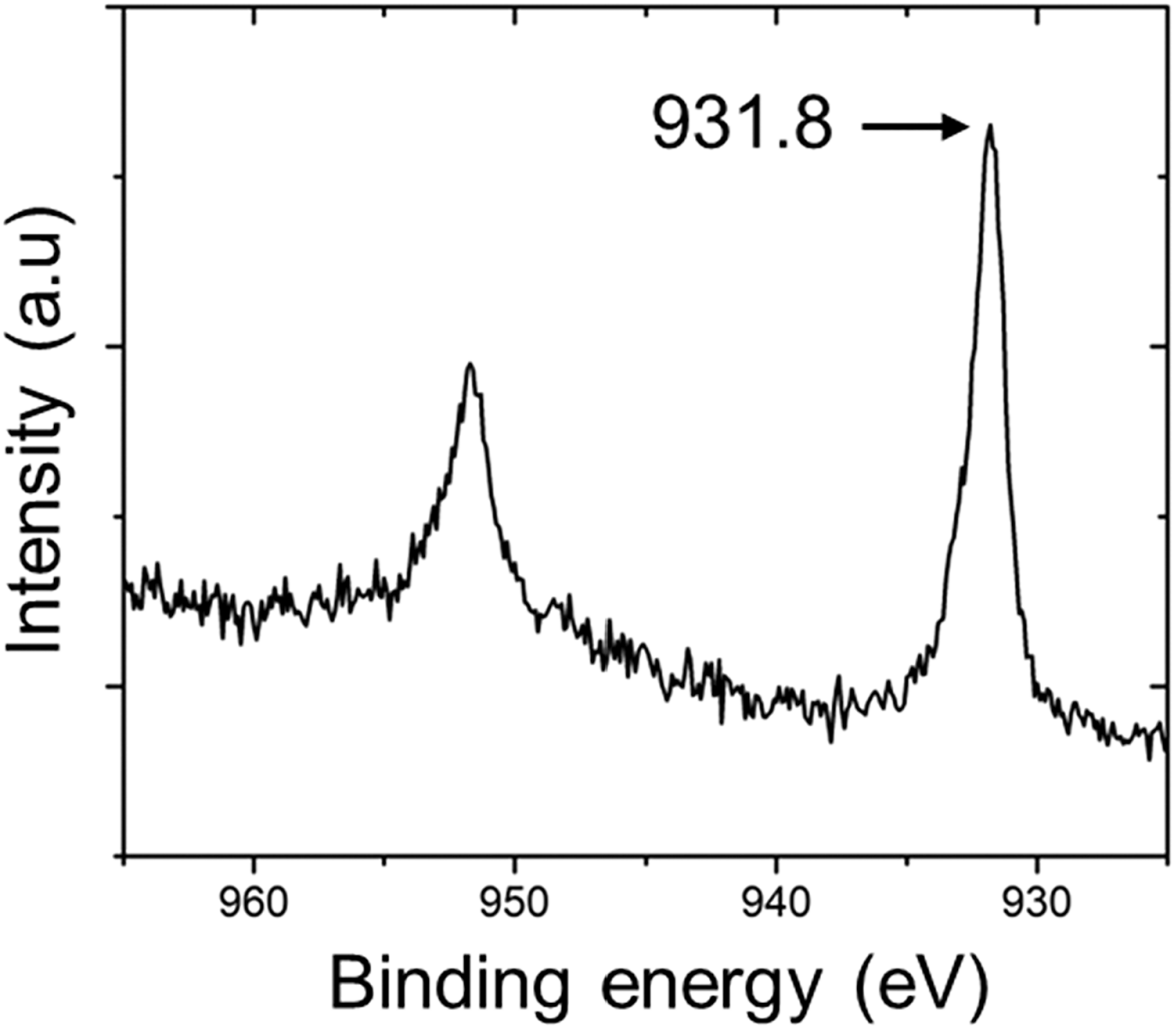
X-ray photoelectron spectroscopy (XPS) spectrum of CuYbSe_2_ highlighting the Cu 2p region.

Although the presence of a structural transition is clearly supported by neutron diffraction, resistivity, and heat capacity measurements, it is less apparent in X-ray diffraction. Temperature-dependent single-crystal X-ray diffraction (SCXRD) measurements were performed, allowing refinement in both the parent *P*-3*m*1 and monoclinic models (Tables S1–S2). Refinement using the monoclinic structure yields a lower goodness-of-fit (GoF ∼ 1.15) compared to the *P*-3*m*1 model (GoF ∼ 1.5), suggesting a better description of the data. The relatively high GoF values in both cases likely arise from residual structural disorder persisting even at 280 K. The *P*-3*m*1 model does not adequately capture the excess electron density arising from displaced Cu atoms, whereas the monoclinic structure accommodates these positional shifts more effectively. Direct comparison of the monoclinic and *P*-3*m*1 refinements using X-ray diffraction alone reveals only subtle structural differences (Tables S4–S6). However, neutron diffraction refinements exhibit more pronounced deviations between the two models (Table S7). This discrepancy likely originates from the dominant X-ray scattering contribution of Yb^3+^, which masks small atomic displacements associated with Cu. In contrast, neutrons provide comparable scattering contrasts for Cu, Se, and Yb, rendering them more sensitive to light-atom displacements. Furthermore, local disorder introduces broad diffuse scattering that can be up to 3 orders of magnitude weaker than the primary Bragg reflections, making it challenging to detect by X-rays, an effect similarly observed in Cu_2_Se.^34^ Neutron diffraction, by comparison, is more sensitive to such features, accounting for the improved fit and lower residuals in neutron-based refinements. Another complicating factor is twinning between the parent and distorted structures, induced by Cu-site displacements during the phase transition. Mode analysis using ISODISTORT^25,26^ indicates only minor differences in the calculated diffraction patterns between the two space groups, which can easily be obscured by the strong scattering of Yb^3+^. Additional evidence for Cu disorder is observed in the refinement of the monoclinic X-ray data, where continuous shifts of the Cu atomic positions are consistent with dynamic or static site disorder within the lattice.^39^

In addition, ytterbium typically exists in the trivalent oxidation state (Yb^3+^), although the divalent state (Yb^2+^) can be stabilized under specific chemical environments. Similarly, copper exhibits multiple stable oxidation states, most commonly Cu^+^ and Cu^2+^. Simple redox potential considerations suggest that the reduction of Yb^3+^ to Yb^2+^ coupled with the oxidation of Cu^+^ to Cu^2+^ is not thermodynamically favorable under standard conditions; however, such redox processes may occur locally under suitable structural or electronic perturbations. X-ray photoelectron spectroscopy (XPS) measurements confirm that copper predominantly adopts the Cu^+^ oxidation state under ambient conditions, as evidenced by the absence of the characteristic Cu^2+^ satellite feature in the Cu 2p_3/2_ region.^40^ The observed lower binding energy indicates that the electronic environment facilitates partial delocalization of Cu electrons and may stabilize the resulting hole states. Notably, both the scanning electron microscopy (SEM) and XPS analyses exhibit no charging artifacts, implying a sufficient degree of electrical conductivity within the crystal to dissipate surface charge, which is consistent with a lightly doped or defect-compensated semiconducting behavior rather than a fully insulating one.

## Conclusions

In summary, we have investigated the structural transition and transport properties of CuYbSe_2_. Single-crystal X-ray diffraction identifies a *P*-3*m*1 structure at room temperature. Upon cooling, the diffraction data can be modeled using either the parent *P*-3*m*1 or the distorted monoclinic *I*2/*m* structure. The transition to the *I*2/*m* phase is unambiguously supported by heat capacity, electrical resistivity, and neutron powder diffraction measurements. The absence of a distinct signature in single-crystal X-ray diffraction likely arises from the inherently weak diffuse scattering associated with Cu-site disorder, which is overshadowed by the intense Bragg reflections, a phenomenon also observed in the low-temperature β-Cu_2_Se phase.

## Supplementary Material



## Notes

SEM pictures, EDS analysis, temperature-dependent single crystal X-ray diffraction refinements, neutron diffraction refinements, structural evolution (PDF)
