# Supplementary material for: Temperature-Dependent Structural Transition in Cu-Intercalated Trigonal CuYbSe2
Source: Chem Mater. 2025 Dec 2;38(1):328–35. doi: 10.1021/acs.chemmater.5c02470 (PMC12805515; doi:10.1021/acs.chemmater.5c02470)
Supplement: Supplementary file 1 [file cm5c02470_si_001.pdf]

Supporting Information

Temperature-dependent Structural Transition in Cu-intercalated Trigonal $CuYbSe_2$

Matt Boswell[1,2], Mingyu Xu[1], Saban M. Hus[3], Antonio M. dos Santos[2], Weiwei Xie[1*]

1. Department of Chemistry, Michigan State University, East Lansing, MI 48824 USA
2. Neutron Scattering Division, Oak Ridge National Laboratory, Oak Ridge, TN 37831 USA
3. Center for Nanophase Materials Sciences, Oak Ridge National Laboratory, Oak Ridge, TN 37831 USA

Corresponding author: Weiwei Xie (xieweiwe@msu.edu)

Table of Contents

Table S1. Temperature dependent single crystal structure refinement parameters (P-3m)--------P2

Table S2. Temperature dependent single crystal structure refinement parameters (I2/m)---------P2

Table S3. Temperature dependent powder neutron diffraction structure parameters--------------- P3

Table S4. P-3m crystal atomic coordinates and Wyckoff sites---------------------------------------P3

Table S5. I2/m crystal atomic coordinates and Wyckoff sites---P3

Table S6. C2/m crystal atomic coordinates and Wyckoff sites--P3

Table S7. I2/m crystal atomic coordinates from neutron powder diffraction------------------------P4

Fig S1. SEM-EDS of $CuYbSe_2$--P4

Fig S2. $CuYbSe_2$ C2/m structure with disorder--- P5

Fig S3. Comparison between P-3m and I2/m structures--P5

Table S1. Temperature dependent single crystal measurement fit with the P-3m1 space group.

Temperature	300 K	280 K	260 K	240 K	220 K	200 K	180 K	140 K	100 K
**a (Å)**	4.0247 (1)	4.0252 (1)	4.0244 (1)	4.0229 (1)	4.0214 (1)	4.0183 (1)	4.0174 (1)	4.0140 (1)	4.0129 (1)
**b (Å)**	4.0247 (1)	4.0252 (1)	4.0244 (1)	4.0229 (1)	4.0214 (1)	4.0183 (1)	4.0174 (1)	4.0140 (1)	4.0129 (1)
**c (Å)**	6.4444 (4)	6.4444 (3)	6.4428 (2)	6.4401 (3)	6.4358 (2)	6.4353 (3)	6.4326 (2)	6.4302 (2)	6.4246 (2)
**Volume (Å^3)**	90.402 (6)	90.429 (5)	90.367 (4)	90.261 (5)	90.134 (4)	89.988 (5)	89.910 (4)	89.724 (4)	89.597 (4)
**Rint (%)**	8.91	8.67	8.35	8.33	8.50	8.79	8.86	8.67	9.10
**R1/ wR2 (%)**	4.62/ 11.42	5.61/ 13.03	5.82/ 13.42	5.76/ 13.35	5.72/ 12.67	5.72/ 12.93	5.84/ 12.75	5.58/ 12.71	5.57/ 12.61
**GoF (%)**	1.085	1.587	1.525	1.578	1.550	1.552	1.5639	1.571	1.526
**Peak/hole**	9.9/ -3.1	11.3/ -5.4	10.6/ -5.6	10.0/ -5.7	11.3/ -5.6	11.0/ -5.6	11.8/ -5.8	10.6/ -7.6	10.9/ -7.2

Table S2. Temperature dependent single crystal measurement fit with the I2/m space group

Temperature	280 K	260 K	240 K	220 K	200 K	180 K	140 K	100 K
**a (Å)**	6.9718 (3)	6.9714 (3)	6.9664 (3)	6.9645 (3)	6.9578 (3)	6.9550 (3)	6.9528 (3)	6.9480 (3)
**b (Å)**	4.0254 (2)	4.0232 (2)	4.0231 (2)	4.02017 (2)	4.0179 (2)	4.0171 (2)	4.0135 (1)	4.0135 (2)
**c (Å)**	12.8864 (6)	12.8826 (5)	12.8776 (6)	12.8702 (5)	12.8699 (5)	12.8666 (5)	12.8594 (5)	12.8473 (5)
**β**	90.010 (4)	90.022 (3)	90.024 (4)	90.010 (4)	90.001 (4)	89.996 (4)	90.007 (3)	90.015 (3)
**Volume (Å^3)**	361.65 (3)	361.33 (3)	360.91 (3)	360.39 (3)	359.79 (3)	359.48 (3)	358.84 (2)	358.269 (3)
**Rint (%)**	7.52	7.78	7.01	7.24	7.97	7.39	7.59	7.92
**R1/ wR2 (%)**	6.38/ 17.38	6.49/ 17.62	6.66/ 19.18	6.49/ 17.94	7.06/ 17.56	6.83/ 17.05	6.79/ 16.63	6.91/ 17.37
**GoF (%)**	1.121	1.150	1.175	1.142	1.242	1.143	1.131	1.181
**Peak/hole**	8.3/ -6.2	10.4/ -5.5	7.3/ -6.5	7.7/ -6.5	10.8/ -6.1	8.8/ -6.6	10.8/ -7.6	9.7/ -7.9

Temperature	290 K	246 K	192 K	135 K	112 K	87 K	80 K
a (Å)	4.026 (1)	6.940 (5)	6.933 (4)	6.925 (4)	6.921 (3)	6.927 (5)	6.924 (4)
b (Å)	4.026 (1)	4.030 (3)	4.025 (2)	4.026 (2)	4.025 (2)	4.020 (3)	4.017(2)
c (Å)	6.453 (2)	12.900 (5)	12.892 (5)	12.886 (4)	12.878(5)	12.875 (5)	12.868 (4)
β	90	90.14 (8)	90.10 (8)	90.16 (7)	90.15 (7)	90.13 (9)	90.07 (8)
Volume ($Å^3$)	90.57 (2)	360.7 (1)	359.8 (1)	359.3 (1)	358.8 (1)	358.5 (1)	358.0 (1)

Table S3. Neutron powder diffraction refined parameters for both the *P-3m* space group (290 K) and the *I2/m* space group.

Atom	x	y	z	Occ	Site	Sym
Yb	0	0	0	0.978	1a	-3m
Cu	0.3333	0.6667	0.24710	1	2d	3m
Se	0.3333	0.6667	0.6206	0.54	2d	3m

Table S4. Single crystal X-ray diffraction atomic positions and occupancies for $CuYbSe_2$ in the *P-3m* space group

Atom	x	y	z	Occ	Site	Sym
Yb1	0	0	0	0.922	2a	2/m
Cu1	0.3327	0	0.3098	0.592	4i	m
Se3	0.3334	0	0.12356	1	4i	m
Yb2	0	0	0.5	0.965	2a	2/m
Cu2	0.16550	0.5	0.6893	0.589	4i	m
Se4	0.1665	0.5	0.87646	1	4i	m

Table S5. Single crystal X-ray diffraction atomic positions and occupancies for $CuYbSe_2$ in the *I2/m* space group

Atom	x	y	z	Occ	Site	Sym
Yb1	0	0.5	0	0.922	2b	2/m
Cu1	0.1779	0	0.0449	0.592	4i	m
Se3	0.1183	0	0.30430	1	4i	m
Yb2	0	0	0.5	0.965	2c	2/m
Cu2	0.299	0	0.434	0.589	4i	m
Se4	0.3729	0	0.20690	1	4i	m

Table S6. Single crystal X-ray diffraction atomic positions and occupancies for $CuYbSe_2$ in the C2/m space group. C2/m is the standard setting for the I2/m space group.

Atom	x	y	z	Occ	Site	Sym
**Yb1**	0	0	0	0.922	2a	2/m
**Cu1**	0.36879	0	0.30855	0.910	4i	m
**Se3**	0.31502	0	0.11623	1	4i	m
**Yb2**	0	0	0.5	0.965	2a	2/m
**Cu2**	0.14685	0.5	0.68117	0.350	4i	m
**Se4**	0.18378	0.5	0.87803	1	4i	m

Table S7. Neutron powder diffraction atomic positions and occupancies for $CuYbSe_2$ in the *I2/m* space group at 80 K.

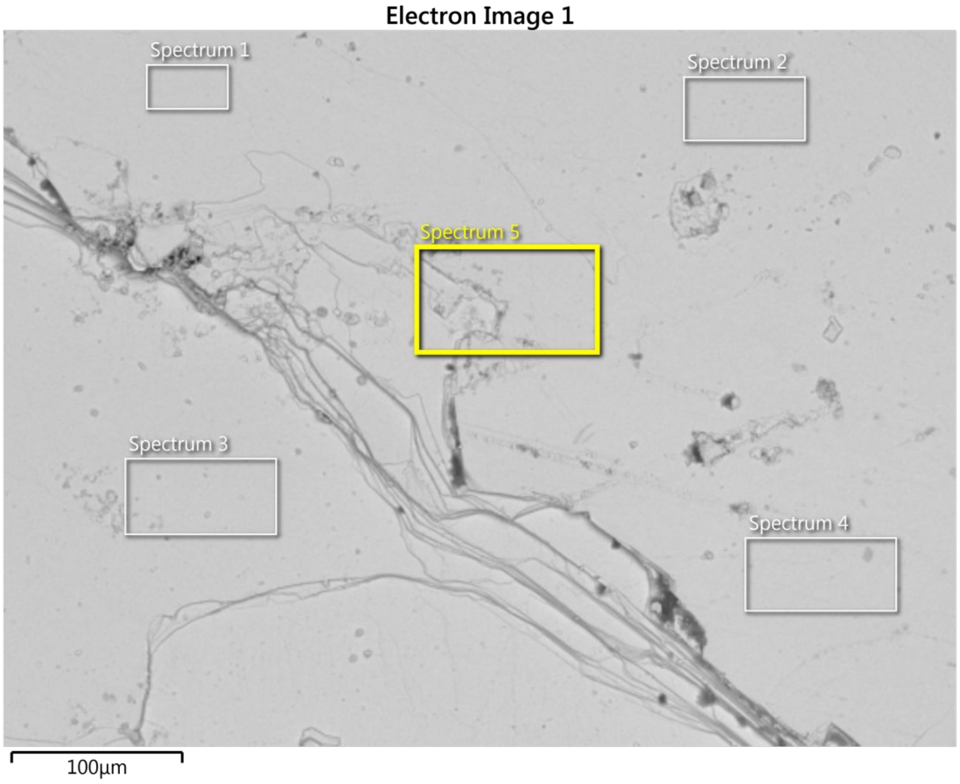

Se	49.66%
Yb	24.68%
Cu	23.72%
O	2%

Fig S1. SEM-EDS scan of $CuYbSe_2$ and averaged values from the 5 spectra taken.

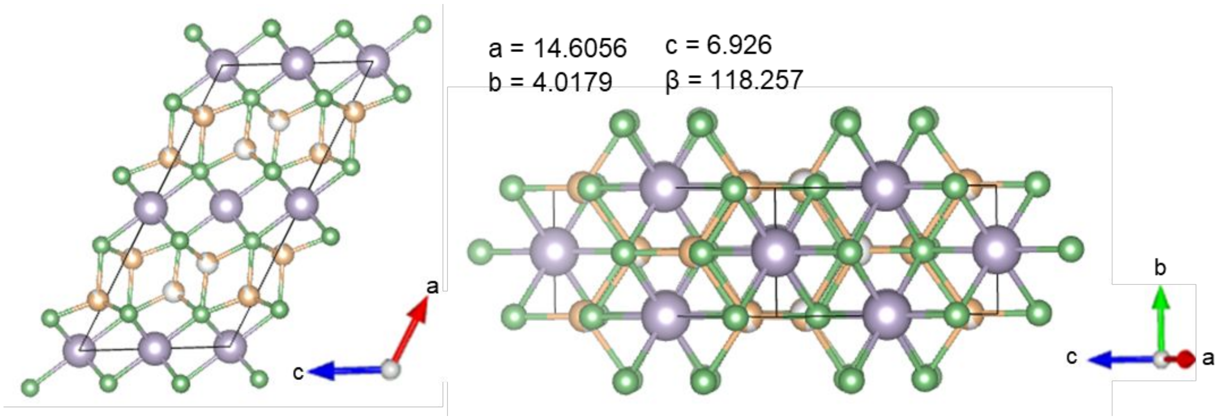

Fig S2. $CuYbSe_2$ in the *C2/m* space group, the standard setting for the *I2/m* space group.

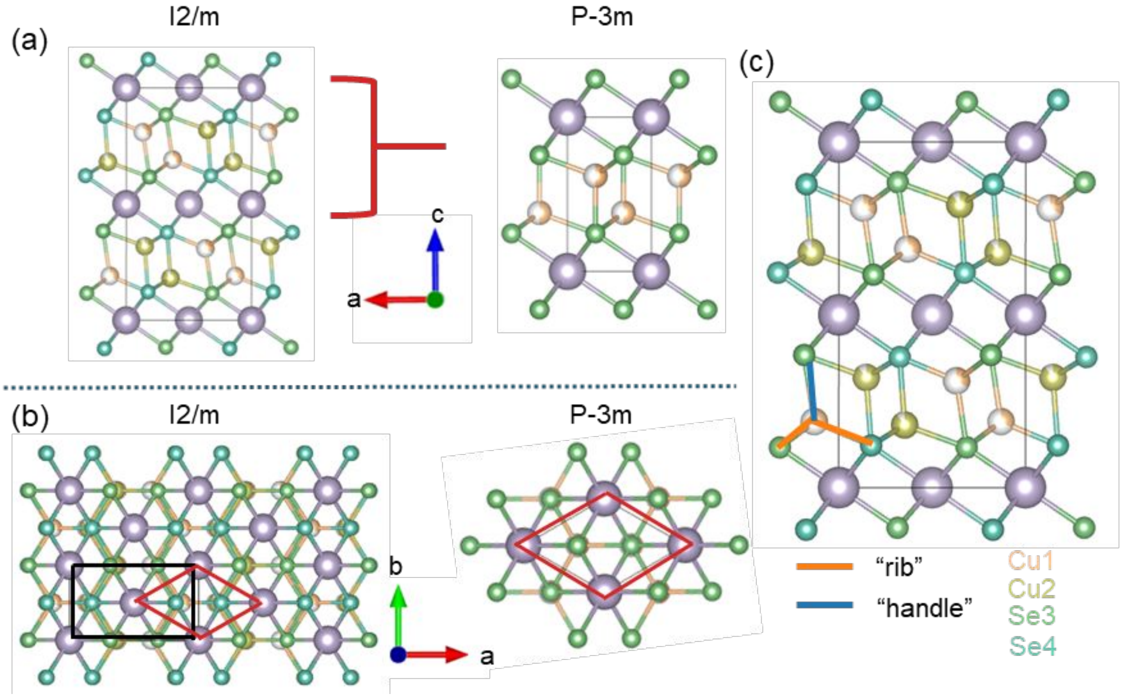

Fig S3*a*. Comparison between monoclinic low temperature structure to the P-3m parent structure looking down the b-axis. **(*b*)** Comparison between the monoclinic and trigonal structure looking down the c-axis. Black box indicates one unit cell for the monoclinic structure while the red box is on unit cell for the trigonal structure. **(*c*)** Monoclinic structure with Se and Cu sites labeled.
